# Progress of Epidemics in Forty-Two Towns and Districts

**Published:** 1859-01

**Authors:** 


### PROGRESS OF EPIDEMICS.

### LOCAL REPORTS OF EPIDEMIC AND ENDEMIC DISEASES

*During the Months of September, October, and November,* 1858.

| Place. | County. | Lat. | Long. | Observer. |
|---|---|---|---|---|
| St.Mary, Scilly | Cornwall - | 49.50 N. | 6.24 W. | J. G. Moyle, Esq. |
| Teignmouth - | Devonshire - | 50.32 N. | 3.29 W. | W. C. Lake, Esq. |
| Portsmouth - | Hampshire - | 40.58 N. | 6. 6 W. | Alfred Jackson, M.D. |
| Odiham - - | Hampshire - | 51. 8 N. | 1. 3 W. | J. McIntyre, M.D. |
| Canterbury - | Kent - - | 51.17 N. | 1. 4 E. | G. Rigden, Esq. |
| Chatham - | Kent - - | 51.21 N. | 0.14 E. | F. J. Brown, M.D. |
| Wandsworth - | Surrey - - | 51.28 N. | 0. 7 W. | G. E. Nicholas, Esq. |
| Putney - - | Surrey - - | 51.28 N. | 0. 8 W. | R. H. Whiteman, Esq. |
| Up. Holloway | Middlesex - | 51.32 N. | 0.03 E. | W. B. Kesteven, Esq. |
| Hawkesbury - | Gloucestersh. | 51.34 N. | 3.20 W. | W. I. Cox, Esq. |
| Swansea - | Glamorgansh. | 51.38 N. | 2.50 W. | W. H. Michael, Esq. |
| Colchester - | Essex - - | 51.54 N. | 0.52 E. | Henry Laver, Esq. |
| Aspley Guise - | Bedfordshire - | 52. 1 N. | 0.37 W. | J. Williams, M.D. |
| Saffron Walden | Essex - - | 52. 3 N. | 0.12 E. | T. Spurgin, Esq.<br>H. Stear, Esq. |
| Bedford - - | Bedfordshire - | 52. 8 N. | 0.51 W. | T. H. Barker, M.D. |
| Newpt.Pagnell | Buckinghams. | 52.10 N. | 0.42 W. | G. O. Rogers, Esq. |
| Sharnbrook - | Bedfordshire - | 52.12 N. | 0.40 W. | R. S. Stedman, Esq. |
| Wellingbro' - | Northamptons. | 52.20 N. | 0.40 W. | B. Dulley, Esq. |
| Beccles - - | Suffolk - - | 52.25 N. | 1.48 E. | W. E. Crowfoot, Esq. |
| Thetford - | Norfolk - - | 52.26 N. | 0.45 E. | H. W. Bailey, Esq. |
| Stourbridge - | Worcestersh. - | 52.28 N. | 2.32 W. | A. Freer, Esq. |
| Dudley - - | Staffordshire - | 52.30 N. | 2.10 W. | J. H. Houghton, Esq. |
| Barrowden - | Rutland - | 52.34 N. | 0.38 W. | H. J. Swann, Esq. |
| Wisbeach - | Cambridgesh. | 52.39 N. | 0. 5 E. | W. H. Hole, Esq. |
| East Dereham | Norfolk - - | 52.40 N. | 0.57 E. | J. Vincent, M.D. |
| Pontesbury - | Shropshire - | 52.43 N. | 2.50 W. | Wm. Eddowes, Esq. |
| Nottingham - | Nottinghamsh. | 52.50 N. | 1.10 W. | T. Robertson, M.D. |
| Burton-on-Trt. | Staffordshire - | 52.53 N. | 1.53 W. | S. Thomson, M.D. |
| Oswestry - | Shropshire - | 52.53 N. | 2.59 W. | P. Cartwright, Esq. |
| Wrexham - | Denbighshire - | 53. 2 N. | 3. 1 W. | E. Williams, M.D. |
| Hawarden - | Flintshire - | 53.11 N. | 3. 2 W. | T. Moffat, M.D. |
| Lincoln - | Lincolnshire - | 53.12 N. | 0. 5 W. | S. Lowe, Esq. |
| Alford - - | Lincolnshire - | 53.15 N. | 0. 6 E. | R. U. West, M.D. |
| Gainsborough | Lincolnshire - | 53.23 N. | 0.47 W. | D. Mackinder, M.D. |
| Liverpool - | Lancashire - | 53.24 N. | 2.59 W. | Thos. Bickerton, Esq. |
| Warrington - | Lancashire - | 53.25 N. | 2.32 W. | C. N. Spinks, Esq. |
| Wigan - - | Lancashire - | 53.32 N. | 2.33 W. | Jephtha Hussey, Esq. |
| Bolton - - | Lancashire - | 53.35 N. | 2.19 W. | W. H. Pendlebury, Esq. |
| York - - | Yorkshire - | 53.58 N. | 1. 3 W. | W. Procter, Esq. |
| Wst. Auckland | Durham - | 54.45 N. | 1.40 W. | G. Todd, Esq. |
| Rothbury - | Northumberld. | 55.25 N. | 1.50 W. | E. C. Summers, Esq. |
| Lerwick - | Shetland Isles | 60. 9 N. | 1. 8 W. | G. W. Spence, M.D. |

### QUARTERLY STATEMENT—No. XVI.

[*The dates denote that the disease appeared in the weeks then ending.*]

SCARLET FEVER.

Teignmouth—Sep. 10-24, all Oct. and [Nov.
Portsmouth—Nov. 26
Canterbury—Sep. 3-17
Chatham—Sep. 17, Oct. 8-15, Nov. 12
Wandsworth—Oct. 8, 22-29, all Nov.
Putney—Sep. 3, Oct. 1-8, 29, all Nov.
Up. Holloway—Every week
Hawkesbury Upton—Sep. 10-24, all [Oct. & Nov.
Swansea—Every week

Saffron Walden—Nov. 26
Sharnbrook—Every week
Wellingborough—Every week
Beccles—Sep. 3-24, Oct. 1-15
Thetford—Sep. 3-17, Oct. 22-29, Nov. 12-26
Stourbridge—Sep. 24, Oct. 1-8, 29, Nov. 5-19, 26
Dudley—Sep. 3-10, Oct. 1, 15, 29, Nov. 5, 19-26
Barrowden—Sep. 3-17, Nov. 5-19
Wisbeach—Oct. 22-29, all November
Pontesbury—Every week
Nottingham—Sep. 3, 17-24, Oct. 8, 22, Nov. 5-19
Burton-on-Trent—November 19
Wrexham—September 10-17
Hawarden—November 5
Gainsborough—Sep. 10-24, Oct. 1 8, 29, [Nov. 5
Liverpool—Every week
Wigan—Sep. 10, 24, Oct. 8, 29, Nov. [12-26
Bolton-le-Moors—Every week
York—Sep. 3, 17-24, Oct. 8, 22, all Nov.
Lerwick—Sep. 10

MEASLES.

Teignmouth—Oct. 1-22, Nov. 5, 19-26
Portsmouth—September 17
Canterbury—Every week
Chatham—September 17, October 1
Up. Holloway—November 19-26
Wanstead—November 12
Swansea—All September, Oct. 1-15
Wellingborough—November 12-26
Thetford—October 15
Stourbridge—All Sep. Oct. 1, 15-29, [Nov. 5-26
Wisbeach—All Nov.
Nottingham—Sep. 3, 17, Oct. 22
Alford—All September, October 1
Liverpool—Every week
Wigan—Oct. 1, 15, 29, Nov. 12, 26
Bolton-le-Moors—Every week
York—Sep. 3, 17-24, Oct. 8, 22, all Nov.

SMALL-POX.

St. Mary's, Scilly—November 26
Portsmouth—October 22
Chatham—November 5-12
Up. Holloway—Sep. 24, Oct. 1
Swansea—Oct. 1, 29
Aspley Guise—November 26
Bedford—Oct. 22-29, Nov. 5-12
Dudley—October 29
Barrowden—October 1-8
Nottingham—October 1, Nov. 12, 26
Gainsborough—Every week
Liverpool—Every week
Bolton-le-Moors—Sep. 3-17, Oct. 15-29 [all Nov.
Rothbury—October 1
Lerwick—September 3

HOOPING COUGH.

Teignmouth—Oct. 1-8, 22, Nov. 5, 19
Portsmouth—September 24
Canterbury—September 10
Chatham—September 17, October 1
Putney—October 29, all November
Up. Holloway—September 3
Hawkesbury Upton—Sep. 3-10
Swansea—Sep. 1-10, 29, Oct. 8
Bedford—October 15-29
Beccles—Oct. 22-29, Nov. 5-12
Thetford—October 22
Dudley—November 12
East Dereham—All November
Pontesbury—All September, Oct. 1
Oswestry—Sep. 24, all Oct., all Nov.
Wrexham—Oct. 8-29, all November
Alford—All September, October 1
Gainsborough—All September
Liverpool—Every week
Bolton-le-Moors—Sep. 17-24, Oct. 1, 22-29. Nov. 5-19
York—Sep. 10, Oct. 8-22, all Nov.
Rothbury—September 10-24

CROUP.

Canterbury—November 5
Chatham—October 22
Putney—November 19
Up. Holloway—October 22
Hawkesbury Upton—November 26
Swansea—Sep. 1, 24, October 8
Aspley Guise—September 3-10
Newport Pagnell—Oct. 22, Nov. 26
Beccles—November 12-19
Thetford—September 3
Stourbridge—November 19
East Dereham—October 8
Wrexham—November 5-19
Liverpool—Oct. 22-29, all November
Bolton-le-Moors—Oct. 1-15, Nov. 5-12, [26
York—Oct. 8-29, Nov. 12

CATARRH.

St. Mary's, Scilly—Oct. 8-29, all Nov.
Teignmouth—Oct. 8-22, Nov. 12-26
Chatham—Sep. 17, October 15-22
Wandsworth—Sep. 10-24, Oct. 1, 15-29, [all Nov.
Putney—Every week
Hawkesbury Upton—Oct. 8-22
Swansea—Oct. 8-29, all November
Aspley Guise—November 12-26
Saffron Walden—November 19-26
Bedford—November 12-19
Newport Pagnell—November 19-26
Sharnbrook—Sep. 10-24, Oct. 8-29, all November
Wellingborough—Oct. 15-29, all Nov.
Beccles—Sep. 24, Oct. 1-22, Nov. 12, 26
Thetford—Sep. 3-10, 24, Oct. 1-22, all [Nov.
Stourbridge—Oct. 8-29, all Nov.
Dudley—Sep. 3-10, 24, Oct. 1-8, 22-29, November 26
Wisbeach—Oct. 22 29, all November

East Dereham—All November
Pontesbury—Oct. 8-29, all November
Nottingham—November 5, 19-26
Burton-on-Trent—Nov. 5-19
Oswestry—Every week
Wrexham—Nov. 12-26
Gainsborough—Oct. 8-29, Nov. 5-19
Liverpool—Every week
Wigan—Oct. 15, Nov. 5, 26 [all Nov.
Bolton-le-Moors—Sep.3-17, Oct.15-22,
York—Sep.17, Oct.1,15,29, Nov.5,19-26
Rothbury—November 12-26
Lerwick—Sep. 17, Oct. 15-22, Nov. 12

INFLUENZA.

Teignmouth—Oct. 15, Nov. 12, 26
Chatham—Sep.17-24, Oct.1-8, all Nov.
Saffron Walden—Oct. 29, Nov. 19-26
Bedford—Oct. 22-29, Nov. 5-19
Newport Pagnell—Sep. 3-10, Oct. 8-29,
Sharnbrook—Nov. 12-26 [all Nov.
Wellingborough—Nov. 12-26
Beccles—Oct. 29, Nov. 5 [19-26
Thetford—Sep. 10-17, Oct. 8-22, Nov.
Stourbridge—Oct. 15-29, all Nov.
East Dereham—All November
Pontesbury—Oct. 29, Nov. 5-19
Nottingham—Sep. 24, Oct. 8
Oswestry—October 29, all November
Wrexham—Nov. 12-26
Hawarden—All November
Liverpool—All Sep., all Oct., Nov. 5-12
Wigan—October 8
Bolton-le-Moors—All Oct., all Nov.
York—Sep.3,17, Oct.8,29, Nov.5-12,26
Lerwick—Oct. 15-29, Nov. 5-19

ERYSIPELAS.

Teignmouth—November 26
Southsea—Oct. 22, Nov. 19
Canterbury—October 29
Chatham—Sep. 3, 17, Oct. 8, Nov.12-26
Wandsworth—October 1-8, 22
Putney—September 3, 24, October 1
Wanstead—October 29, Nov. 5-12
Swansea—September 10
Saffron Walden—October 15
Bedford—September 17 [Nov.12-26
Newport Pagnell—All Sep. Oct. 1, 22,
Wellingborough—Oct. 22-29, Nov. 19-
Beccles—Oct. 8-15, Nov. 26 [26
Stourbridge—Sep. 3, 24, Nov. 19
Dudley—September 17-24, October 1
Barrowden—October 29
Wisbeach—September 17-24, Nov. 12
East Dereham—September 3-10
Pontesbury—Sep. 17-24, Oct. 1, 22-29,
November 19-26
Nottingham—Oct. 1, 22, Nov. 12, 26
Oswestry—All November
Hawarden—Oct. 8-15
Gainsborough—Oct. 8-29, Nov. 19-26
Liverpool—Every week
Wigan—Oct. 22, Nov. 5, 19
Bolton-le-Moors—Sep. 17, Oct. 15, 29,
York—Sep. 17, Oct. 15 [Nov. 5-12
Lerwick—October 29

AGUE.

Southsea—September 17
Canterbury—All Sep., Oct. 8-22
Chatham—October 8-15, Nov. 19-26
Bedford—October 1-8
Wellingborough—All Oct. and Nov.
Beccles—September 17-24, October 1
Thetford—September 3, Nov. 12-26
Nottingham—October 8
Stourbridge—November 26
Barrowden—Sep. 3-10, Oct. 15, Nov. 12
Wisbeach—Sep. 10-24, Oct. 1, 22-29,
East Dereham—Sep. 3-10 [Nov. 12
Liverpool—November 19-26

REMITTENT FEVER.

Teignmouth—Sep.17, Oct.29, Nov.12
Canterbury—September 10, 24
Putney—September 24, November 5
Wanstead—October 8-15
Newport Pagnell—All Sep., Oct. 1-22
Beccles—November 5-19 [19-26
Thetford—All Sep., Oct. 8-15, 26, Nov.
Stourbridge—September 3
Oswestry—September 3-17, Nov. 26
Alford—All September, October 1
Liverpool—Sep. 3-10, Oct. 1, 22, Nov.
Wigan—Oct. 1, 15, Nov. 12 [12-26
Bolton-le-Moors—Every week
York—Sep. 24, Oct. 15, 29

DIARRHŒA.

Teignmouth—Sep.10-24, all Oct., Nov.
Southsea—Sep. 3, 17, Oct. 1-8 [5-26
Canterbury—All September, Nov. 5-12
Chatham—Sep. 3-10, Oct. 1, 15-29,
November 12-26
Wandsworth—All Sept. & Oct., Nov.
Putney—All Sep. and Oct. [19-26
Hawkesbury Upton—All September
Swansea—All September, October 1
Saffron Walden—Sep. 10-17, Oct. 1,
15-22, November 19-26
Bedford—September 3-10, October 22
Newport Pagnell—Every week
Sharnbrook—Sep.17, Oct.1-15, Nov. 5
Wellingborough—All Sept. and Oct.
Beccles—October 1-8, 22
Thetford—Sep. 3-10, 24, Oct. 1, 15-22,
November 5-19
Stourbridge—All Sep., Oct. 1-22
Dudley—Every week
Wisbeach—All Sept. [Nov 5-19
East Dereham—Sep. 3-10, Oct. 22-29,
Pontesbury—All Sep., Oct. 1-8
Nottingham—Sep. 24, Oct. 1, 15-22
November 12-19

Burton-on-Trent—Sep. 10-24, Oct. 29, [Nov. 5-12
Oswestry—Every week
Wrexham—All October & November
Hawarden—Sep. 3-17, Nov. 19-26
Alford—All Sep., Oct. 1, 29, all Nov.
Gainsborough—Every week
Liverpool—Every week
Wigan—Oct. 15, 29
Bolton-le-Moors—Sep. 3-17, Oct. 1-8
York—Sep. 10, Oct. 1, 22, Nov. 5, 19-26
Rothbury—Oct. 8, 29
Lerwick—Sep. 3-10, Nov. 5, 19

DYSENTERY.

Teignmouth—November 12
Chatham—Sept. 10-17 [Nov. 5
Wandsworth—Sept. 3-10, Oct. 8, 22-29,
Wanstead—Sept. 10-24, Oct. 1-8
Swansea—September 10-17
Saffron Walden—Nov. 26 [12
Newport Pagnell—Oct. 15 22, Nov. 5-
Stourbridge—Sep. 3-10, 24, Oct. 1-8,
East Dereham—Sep. 3-10 [Nov. 12
Nottingham—Sept. 10, Oct. 8, 22-29,
Wrexham—All Oct., Nov. 5-12 [Nov. 12
Gainsborough—Sep. 24, Oct. 1-22, 29,
Liverpool—Oct. 1-22, all Nov. [Nov. 5-19
Bolton-le-Moors—Sep. 10, 24, Oct. 1-8, 22-29

TYPHUS.

Portsmouth—Sep. 3-17, all Oct., Nov.
Canterbury—Every week [19
Chatham—Sep. 3-17, Oct. 1-8, 22
Wandsworth—Sep. 3-17, Oct. 8, Nov.
Putney—Sep. 3, 17, Oct. 15 [5, 26
Hawkesbury Upton—October 1
Bedford—All October
Beccles—October 1-22
Thetford—September 24
Dudley—All Sep., Oct. 1-8, 29, Nov. 5,
Wisbeach—All September [26
Pontesbury—Sep. 17-24, all Oct. & Nov.
Nottingham—Sep. 10-17, Oct. 1
Wrexham—Sep. 17-24, Oct. 1-22
Alford—All Sep., Oct. 1
Gainsborough—October 1
Liverpool—Every week [29, all Nov.
Bolton-le-Moors—Sep. 17-24, Oct. 15-

DIPHTHERIA.

Portsmouth—Sep. 17, Oct. 15, Nov. 5,
Canterbury—Oct. 15 [19-26
Wandsworth—November 5
Alford—Every week

PUERPERAL FEVER.

Newport Pagnell—Sep. 24, Oct. 1-15,
Barrowden—Sep. 10 [Nov. 19-26
Nottingham—Sep. 3, 17, Oct. 8-15
Alford—October 15
Wigan—November 5, 19
Bolton-le-Moors—October 15
Lerwick—September 24

TYPHOID FEVER.

Burton-on-Trent—All Sep. and Oct.,
Wigan—Nov. 5-12 [Nov. 5-19

VARICELLA.

Lerwick—September 24, October 8

GASTRIC FEVER.

Lerwick—October 15

CARBUNCLE.

Canterbury—Sep. 10-17, Oct. 22
Chatham—October 15-22
Putney—September 17-24
Saffron Walden—October 22
Newport Pagnell—Sep. 17-24, Oct. 1
Sharnbrook—Oct. 22, Nov. 12
Wellingborough—October 15-29
Beccles—November 19-26
Thetford—September 17
Stourbridge—October 1-8
East Dereham—September 3-10
Burton-on-Trent—September 10
Oswestry—September 3-17
Gainsborough—September 17-24
Liverpool—November 19-26
Wigan—October 15
Bolton-le-Moors—Oct. 1-15, all Nov.
York—Nov. 5-12, 26
Lerwick—October 15

---

### ADDITIONAL OBSERVATIONS.

*St. Mary's, Scilly.*—Mr. Moyle says: "The quarter has been, on the whole, healthy; the cases being principally those of epidemic catarrh, which disease continued for thirty-three days, during the prevalence of strong easterly winds in October and November. One case of small-pox was landed from a ship in the last week of November: the disease has not spread as yet. The case is doing well. We have had but few deaths during the quarter: three occurred in aged people; one from phthisis; and one from gangrene of the lung. The weather has been fine, though breezy throughout the three months. The depth of rain has been 6·1 inches."

*Teignmouth.*—Mr. Lake writes: "Diarrhœa was not nearly so prevalent as it usually is this quarter. Scarlatina was unusually fatal: the cases that occurred were principally among the lower classes. In most cases, the stage of eruption was passed over well; and the fatality arose from exposure to cold bringing on a renal affection, with concomitant dropsy. If the last quarter (June—August) was painfully healthy for the medical man, this was painfully unhealthy to the public and to himself too."

*Canterbury.*—Mr. Rigden says: "During the three months in this report, the epidemic most prevalent in this city has been measles; several cases of which disease have occurred, but only two have terminated fatally. Continued fever has been frequently met with; and eleven cases have ended in death. Ague has been more common than was the case a few years since; but the disease has generally been quickly cured by large doses of quinine administered immediately before the paroxysm. A few cases of scarlet fever were met with in the early part of September; but this disease is not now prevalent. Two cases of croup terminated fatally in the beginning of November in children aged three years, who had been living in the same neighbourhood. Cases of carbuncle are continually presenting themselves. Diphtheria, which was very prevalent in the early part of the year, and even into the middle of summer, appears now to have almost left us; but one death from this disease, a male aged 28, has been registered during the three months now reported on. The register of deaths for this district shows that in the month of September there were 11 deaths from zymotic diseases; viz., 6 from fever, 1 from measles, 2 from scarlet fever, 1 from diarrhœa, and 1 from asthma. In October, three cases; viz., 2 from fever, and 1 from diphtheria. In November, 6 cases, viz., 3 from fever, 1 from measles, and 2 from croup."

*Chatham.*—Dr. Brown states that cases of nephritis have been extremely numerous during the past three months, Ague has ceased to be prevalent.

*Wandsworth.*—Mr. Nicholas writes: "The most prevalent and severe forms of disease during the past quarter have been those affecting the organs of respiration, and scarlatina. In 358 cases of sickness occurring amongst the poor, the former constituted nearly one-sixth, the latter one-eighth, of the whole. At the period of this return, scarlatina is becoming very general; and, although hitherto of mild character, is assuming a very virulent form of the anginose type. The very varied character in which the disease has presented itself is worthy of remark; in some, slight pyrexia, followed by renal dropsy; in others, slight sore throat with a little febrile disturbance, followed by peeling of the skin; in others, malignant sore throat with abundant gelatiniform exudation, with and without consecutive dropsy, and with and without inflammation of the glands; in one case slight pyrexia and inflamed parotids were the only symptoms (which at any other time I should have named mumps). These varieties, with and without eruption, have occurred, too, under apparently similar circumstances; that is, in different members of the same family in many instances. The sequelæ have been unusually severe; the most common being sloughing of the parotid and cervical glands, and otitis. The tendency to dropsy and the severity of the sequelæ have invariably been inversely as the intensity of the eruption; an effect which might be preconceived from a consideration of the vicarious duties of the skin and kidneys. The indication of treatment, therefore, as regards the circulation, would appear to lie in a carefully sustained diaphoresis."

*Putney.*—Mr. Whiteman says: "Throughout the entire quarter, catarrh has been unusually prevalent. Of the more severe forms of zymotic disease, scarlatina has been the most fatal; but not so much so among the poorer classes as among the classes above them. During the quarter, some cases of low fever, one of which was of a very severe character, occurred in a house situated in a badly drained locality. In the same locality (the property belonging to one of the most stubborn of landlords) cases of diarrhœa were continually coming under treatment in the months of September and October. The water supply to the inhabitants of this neighbourhood is from a well, evidently contaminated by the percolation of soil from a closely approximating cesspool. In the month of September carbuncle showed itself in a very severe form in one or two instances, but the patients, though aged, recovered remarkably well. I have had during the quarter one fatal case of croup in private practice, and in my public practice one of cynanche tonsillaris. The entire absence of small pox, not a single case of which has been treated here for several years past, is a circumstance worthy of record, as well evidencing the prophylactic value of vaccination. As the public vaccinator of the district, I have never depended upon voluntary submission to the operation, but have searched out the unprotected in schools, in public establishments, and amongst the poor, and hence have been able to bring many under its influence who would otherwise have escaped me. To this practice I attribute, in great measure, the immunity of this parish from varioloid disease."

*Hawkesbury Upton.*—Mr. Cox writes: "Scarlatina has been extensively epidemic during the whole quarter. The type has been generally favourable, as will appear from the fact that of 31 cases under my own care but two deaths occurred. The severer cases were in a highly insalubrious and ill-drained portion of the district. The cases of diarrhœa and fever form the subject of a short paper in another part of the SANITARY REVIEW. There is a great scarcity of water, owing to the unusually small amount of rain that has fallen. The temperature was high for the season until November 9th, when severe frost set in with violent easterly gales, which lasted to the 25th, when a thaw occurred. A few scattered cases of pneumonia and 'red water' occurred among cattle in September."

*Swansea.*—Mr. Michael writes: "Two cases of small-pox have fallen under my observation in this town; one imported, and one without any apparent communication with an infected district. Scarlet fever has been unusually prevalent in a very fatal form. Sometimes death has occurred in twenty hours, without the appearance of an eruption, from blood-poisoning; in other cases, the anginose form, with intense coryza, has appeared to cause suffocation without much apparent external swelling of the throat. The rash has, in many cases, assumed the mulberry form, and has been attended at its decline with many eruptions—urticaria, lichen, papular efflorescence, etc. The great and severe changes of weather, often eight to ten degrees in a day, and the prevalence of cold easterly winds at the end of October and November, have led to many fatal cases of internal inflammation from checked transpiration from the skin. Peritonitis, nephritis, and similar affections have carried off many victims. In some parts of the town, the streets have been taken up to lay down a system of drainage, and in many of these parts the mortality has been severe. I am quite unable, however, to trace the mortality to this cause. It has occurred in every district; on the top of the hills surrounding the town, and in its lowest parts. The average number of deaths has been for some time, until the present, 14 per week; but it reached 35 in some of the weeks in November. During the prevalence of cold easterly winds, many sudden deaths occurred, especially from epilepsy and apoplexy. Measles appeared to have entirely disappeared when scarlet fever became more intense."

*Wellingborough.*—Mr. Dulley says: "In the thickly populated and ill-drained village of Earl Barton, scarlet fever has been very prevalent during the whole quarter. Many children have been rapidly carried off by disease more resembling diphtheria than scarlatina maligna; in these cases there has been little or no redness of the skin, but they have commenced with stiffness of the neck, enlargement of the cervical glands followed by ulceration of the tonsils, excoriation of the mouth and lips, which sometimes are covered with aphthæ, shortness of the breath, loss of voice, fœtid breath, etc."

*Thetford.*—Mr. Bailey furnishes the following report for the quarter:

"*September.*—The weather throughout the month was particularly fine and dry. The amount of rain did not exceed three-fourths of an inch. The range of the atmospheric pressure was seven-tenths of an inch, and the prevailing winds were south and west. The temperature was high for the month, the maximum being 78°, and the greater part of the month not lower than 74° in the middle of the day; the evenings were considerably above temperate, or 55°. From the 18th, a considerable reduction of temperature took place, the range of the highest degree being 14°. During this time the greatest quantity of rain fell. The little variation of atmospheric pressure, the dryness of the air and higher temperature than usual, conduced much to the healthiness of the month. Simple scarlatina continued to attack the children in the Union Workhouse: in some cases, swellings of the maxillary glands and anasarca were the result, but no death occurred, nor did the disease extend into the town. A fatal case of croup occurred in a little child who had been attacked some days before medical aid was called in. One case only of ague came under observation. Remittent fever was very prevalent during the month, chiefly amongst the labouring classes, and in two instances in the higher classes. Catarrh and influenza were not so frequent, and in this month I have not known so few cases of diarrhœa. One case of typhus came under treatment; from peculiarity of habit, it became complicated and ran a long course, but ultimately recovered. A most serious case of carbuncle occurred in an elderly man: it was situated in the lumber region, and assumed a deep purple hue and produced extreme constitutional irritation. By early incision, stimulating poultices and tonic treatment, he ultimately did well. With children, I scarcely ever knew so few diseases under notice.

"*October.*—The diagram of the barometer showed great fluctuation for the month, which may, in some measure, be attributed to the force of the winds. The range was 1·2 inch; the prevailing winds south and west. The amount of rain was 1·25 inch, with only four days which were cloudless. The temperature at the maximum was 14° lower than last month. The evenings were cold, and on the night of the 8th a severe frost was experienced. The dew-point nearly equalled the temperature. This change of temperature with moisture gave rise to numerous cases of catarrhal affections and influenza; the latter being complicated with pneumonic symptoms, which in young persons required active treatment; throughout the month it must be considered epidemic. Bronchitic affections among children were more prevalent. Only one case of simple scarlatina occurred in the workhouse, and one case of measles, both of which required but little medical aid. Quinsy was frequently under notice, arising from exposure to the atmospheric changes. Rheumatism, acute and chronic, occurred more frequently during the month. Diarrhœa and remittent fever have lately occurred in four or five cases.

"*November.*—The temperature in this month may be recorded as unusually low, being several degrees below the average; at night, upon the grass, it was observed as low as 10°, and about five feet from the ground at nine o'clock in the morning at 15°. Such a state of temperature I have not observed and recorded for 48 years. The dense fogs, absence of winds, and the extreme cold have been severely felt. The diagram of the temperature has a complete wintry form. The maximum was on the 14th, at 54°; the minimum on the 24th, at 10°; being a range of 44°. The barometrical range during the month was very fluctuating, forming two considerable curves. The amount of rain was only a quarter of an inch; the prevalent winds were north and east. The dew point varied but little from the temperature. Scarlatina now assumes an epidemic character, spreading itself in various parts of the town, with tonsillar and pharyngeal inflammation. In some cases the eruption seems transitory, disappearing suddenly, while the febrile symptoms remain severe and greatest difficulty of swallowing. The disease has attacked a whole family, five in number, one of whom terminated fatally. In a mother and two children, although no eruption was observed, their mouths were covered with a yellow slough, exceedingly offensive, including the greenish tongue, very different to that of scarlatina anginosa, and which I should consider to be cases of diphtheria; their state is now very doubtful. Other cases in the town are also under medical care, and which I consider contagious. Catarrhs are also very prevalent, and remittent fevers still continue under observation. From the extreme cold, children have suffered much from congestions of the lungs, with much febrile action. Rheumatism and sore throat have also been more prevalent. During the extreme cold many cases of diarrhœa occurred. These may be considered as the chief diseases arising from atmospheric vicissitudes. Upon inquiring of our veterinary surgeons, I understand a great mortality has taken place in many dairies from pleuropneumonia. In this locality some farmers have lost seven or eight from the disease. Strangles and fever have also attacked horses, and in some instances proved fatal. Sheep and swine seem to be very healthy.

*Record of Deaths in Twenty-one Parishes (population* 9,574) *from the Registrar's Book.*

| *September.* | | *October.* | | *November.* | |
|---|---|---|---|---|---|
| Typhus .......... | 1 | Consumption ...... | 1 | Diphtheria ........ | 1 |
| Consumption ...... | 1 | Inflammat. of lungs | 1 | Consumption ...... | 3 |
| Croup ............ | 1 | Paralysis .......... | 2 | Paralysis .......... | 1 |
| Disease of heart.... | 1 | Dysentery ........ | 1 | Convulsions ...... | 2 |
| Epilepsy .......... | 1 | Debility .......... | 1 | Disease of liver .... | 1 |
| Convulsions ...... | 1 | Decay of nature .... | 3 | Erysipelas ........ | 1 |
| Nephritis.......... | 1 | | | Childbirth ........ | 1 |
| Atrophy .......... | 1 | | | Debility .......... | 3 |
| Dropsy .......... | 1 | | | Decay of nature .... | 3 |
| Debility .......... | 1 | | | | |
| Decay of nature .... | 3 | | | | |
| Accident .......... | 1 | | | | |
| | 14 | | 9 | | 16 |

*Stourbridge.*—Mr. Freer writes: "The epidemic of scarlatina now prevailing is of a severe type, the throat and glands being, in many instances, badly affected, and a fatal issue not unfrequently resulting. The mucous membrane of the throat is also affected in the vast number of cases of influenza now presenting themselves. On inspection of the fauces, however, but little can be seen beyond slight redness of the tonsils and uvula, this latter being also elongated. The tickling throat cough is often distressing. Two cases of diphtheria of the palate and tonsils, but not extending into the larynx, I attended in the weeks ending November 19 and 26. The first, in a boy aged 5, proved fatal on the 9th day from sheer exhaustion; the tonsils and pharynx became quite rotten, breaking up at the least touch. The second case (in an adult) did well. The characteristic white spot began on the soft palate, and extended thence to all the fauces. The weight of disease seems this quarter to have fallen especially on the throat in various ways."

*Barrowden.*—Mr. Swann says: "Scarlet fever had quite left us, but has again been introduced by a family from

Norwich. I have a few cases of small pox, but very mild. By care and threats I manage generally to confine it to the house where it first makes its appearance. I hear that at Oakham, our county town, it is very prevalent, as many as 150 cases lying at one time. I have had a remarkable case of erysipelas in a child about 2 years old; it began in the right foot, and gradually spread upwards to the loins, then gradually passed down the left leg to the foot, and then disappeared, leaving its usual consequences—œdema and discoloration."

*Pontesbury.*—Mr. Eddowes writes: "I have had four cases of typhus this quarter in three different districts, two miles apart. The first case was in a remarkably clean cottage, about a quarter of a mile distant from any other house, on high ground. The only inmates were a man and his wife, both strong and healthy people, between 40 and 50 years of age. They have one daughter, who is married, and lives at a distance. The wife was taken ill in September. On my first visit, as I went to the house, I observed a very offensive smell; it was less offensive when I had entered the house. I found her in bed with typhus. I immediately began to look about for the cause of the abominable smell, and soon found it. A few yards from the door was a stone cistern covered over with boards. I went to see what it contained. When I was within two yards or so of it, the smell became so intolerable that I stood, and having a stick in my hand, reached out and pushed off one of the boards. It was quite full of pig wash, which had been accumulating during the greater part of the summer; the thinner part had evaporated or drained away, it was of the consistence of grease or thick cream. The upper two inches were a mass of living maggots. I told the man that the stinking was the cause of his wife's illness, and that he must have it removed immediately. He said, 'I put my spade into it one day, and found about two inches of maggots on the top and some potatoe peelings quite fresh down about the middle of it.' The second and third cases were from filth and poor living; they occurred in a house where typhus had been twice before within the last 20 years. The last case I could not at first account for; it was in a very filthy neighbourhood, and near to a cottage where I had had a fatal case of diphtheria a short time before. On my first visit to the child with diphtheria I remarked to the mother that I smelled something very disagreeable. I found the stye at the end of the cottage, in a very filthy state, and a fat pig in it. The stye was a few inches above the floor of the cottage."

*Nottingham.*—Dr. Robertson writes: "Scarlet fever has been by far the most prevalent of the eruptive fevers during the quarter. Not only has the fever itself in its malignant form carried off great numbers of children, but "scarlatina faucium," as it is called by the older writers, in which the throat is affected and not the surface, has been widely spread over Nottingham and the surrounding district. Many cases of this affection, in which careful searching for two or three successive days would have discovered a slight eruption, have been confounded with diphtheria. This latter disease has assumed generally the characters presented in most of the reported cases elsewhere,—a leather-like exudation on the tonsils and sometimes the whole of the throat visible to the eye, and a red glazed appearance of the mucous membrane when this exudation was coughed up or removed. I have had in the hospital several cases of small pox. In one girl it was combined with scarlet fever (for which her medical attendant was treating her when he observed the small pox vesicle appear). In another case, the patient had been twice vaccinated, and had suffered from the disease before. Dysentery is much less prevalent than in the corresponding quarter of last year."

*Oswestry.*—Mr. Cartwright states that intermittent neuralgia has been very prevalent during the last fortnight of November.

*Gainsborough.*—Dr. Mackinder writes: "Had it not been for a fresh visitation of throat affection and the rapid increase of small pox, the past quarter would have been a remarkably healthy one, there having been much less than the usual amount of diseases incidental to the autumnal season. From diphtheria several deaths have occurred in the neighbourhood; one of which happened to a patient of my own, a young, vigorous woman, daughter of a farmer, to whom I was not called until asphyxia was impending. The particulars of this and the other cases of throat affection which have been under my care during the quarter, have been communicated to the Epidemiological Society. The small pox, which was introduced from Walesby on the 24th of July last, is now epidemic, scores of cases being in the town; some very mild, others severe. Eight deaths have been registered in the quarter. Of these, four had not been vaccinated; three were vaccinated in the stage of variolous incubation; and one, aged 45, had been vaccinated or inoculated in infancy. The *three* vaccinated were children, members of a family consisting of four. One of these four children had been vaccinated some time previously, was the first to contract the disease, and had it rather severely, but made a favourable convalescence. On the 2nd

September, three weeks afterwards, the other children were vaccinated, all successfully. On the following day variolous papulæ appeared on one of them; on the seventh day the disease was manifest in a second; and on the eighth day the third child bore unmistakeable evidence of the same affection. All became confluent; all had pulmonary complication; all died. The fact of the vaccination passing through its normal stages uninfluenced by the virus of small pox is worthy of notice. A woman, aged 50, who says she had a severe attack of small pox when 7 years old, is now suffering from that disease. In a yard, where the sunbeams seldom penetrate, and the zephyr never finds an entrance, a horrid, stinking, humanity-disgracing hole, having therein some sixteen habitations, I had under treatment, at one time, sixteen cases of small pox. Two deaths occurred in this place, being unvaccinated cases; and the worst case I ever saw after vaccination here passed through its various stages to health. Though there have been many very mild and modified cases of variola after vaccination, there have been more than a few of the confluent kind, where the vestiges of the Jennerian prophylactic were demonstrably evident. Some very mild cases have happened to the unvaccinated. Though my own opinion of the modifying influence of vaccination has not diminished, the prejudice entertained by a large body of the public will not, I fear, be attenuated by recent experience; the percentage of confluent cases being too great to foster confidence in the protective agency of the vaccine lymph. Re-vaccination has become general, and, as yet, with the best results; no re-vaccinated person having, within the range of my observation, been attacked with small pox. Two or three months ago, I requested the attention of the Board of Guardians to the subject of vaccination and exposure of variolous patients; but my warning was unheeded until the disease had gained an entrance into half the dwellings of the town! The door was ordered to be locked after the horse had escaped. Though we are better off than many localities, in reference to vaccination, that boon to humanity is not nearly so popular as it ought to be. Quackery is rampant in Lincolnshire. Homœopathy, hydropathy, Mesmerism, Coffinism, bone-setting, *et hoc genus omne*, have their numerous admirers, and luxuriate in their ill-gotten gains; but science is dormant, bloodless, and cold, pining under an extinguisher of ferruginous mould, longing for liberty and yearning to do good. 142 pauper patients have been attended in the quarter; 35 in September; 66 in October; and 41 in November;—55 in excess of the previous quarter. Of these 142, twenty were between 60 and 70 years old; sixteen between 70 and 80 years old; and one between 80 and 90 years old. Small pox has not yet got possession of the workhouse, an establishment remarkable for its salubrity at all times."

The following meteorological report has been furnished by Mr. Dyson: "The weather was more unsettled last quarter than in the previous eight months of the year, and the principal rain-fall of the year was in September (2·46 inches) and in October (3·89 inches). An unusual amount of fog prevailed at intervals throughout. Ozone was abundant, as will be observed on reference to the accompanying table, all September, and less in the following months. The barometric pressure was unusually high at the close of October, the maximum being more than 30½ inches; on the 28th November it had fallen to 29·092 inches, accompanied by continued rain. The daily record is contained in the table."

*Hindley in Wigan.*—Mr. Hussey writes—"Phlegmonous erysipelas, puerperal diseases (which have been fatal in many instances), measles, and scarlet fever with bronchial affection, have very much prevailed during the latter end of the quarter ending with November. During the long and sharp frost of late, there was a remarkable increase of the above diseases; and since the change in the weather, zymotic diseases have doubled. The wind during the former period was of an easterly character—due east, north east, and for a time south east. During the latter period it has varied much; but has been principally from the west. Throat affections are rife just now, but of a mild character in this quarter. I have had three cases of tonsillitis in a family of three children—a circumstance I never before witnessed. In neither has there been any rash or strawberry tongue, or anything to indicate scarlatina—no desquamation even. In a sanitary point of view, it is remarkable to find erysipelas and puerperal diseases prevailing at one and the same time. The latter are fearfully on the increase, and there can be no question as to the identity of the two poisons. Erysipelas has crept into one of our neighbouring hospitals, and some of the patients who could do so have returned home, *pro tempore.* I administered a vermifuge to a child lately, in a pauper family, and the result was forty-two round worms, twenty-nine of which I counted myself. The water used for cooking comes from a pit in a field. An infant, eight months old, in the same family, has passed two round worms of late."

*York.*—Mr. Procter writes:—"September and October were

*Township of Gainsborough* (*population in* 1851, 7,261). *Deaths from Aug.* 30*th to Nov.* 26*th*, 1858, *inclusive.*

| Date. | Age. | Sex. M. | Sex. F. | Occupation. | Cause of Death. |
|---|---|---|---|---|---|
| Aug.30 | 27 yrs | — | 1 | D. of labourer | Gastro-enteritis |
| Sep. 14 | 61 „ | 1 | — | Labourer | Chronic peritonitis |
| „ | 3 „ | — | 1 | D. of labourer | Variola |
| 17 | 1 „ | 1 | — | S. of labourer | „ 10 days, diarrhœa 9 days |
| 19 | 7 „ | 1 | — | S. of labourer | Marasmus 3 years and 9 mos. |
| „ | 5 „ | 1 | — | S. of labourer | Variola 22 days, diarr. 2 days |
| 21 | 3 „ | 1 | — | S. of charwoman | Hooping cough, pneumonia |
| 27 | 24 „ | 1 | — | Journey. blacksmith | Phthisis |
| 25 | 46 „ | — | 1 | W. of bricklayer | Dropsy* |
| 26 | 45 „ | 1 | — | Rag gatherer | Chronic bronchitis with emphysema |
| 28 | 54 „ | — | 1 | Widow of seaman | Cirrhosis of liver 4 years, ascites anasarca 2 months |
| Oct. 4 | 10 „ | 1 | — | S. of brickl. labourer | Diphtheria 6 days, cynanche maligna 3 days |
| 1 | 20 „ | — | 1 | D. of farmer | Phthisis pulmonalis 18 mos. |
| 3 | 55 „ | — | 1 | W. of gardener | Valv. dis. of heart 2 yrs., bronchitis |
| 7 | 30min | — | 1 | D. of labourer | Premature birth |
| 11 | 1 yr. | 1 | — | S. of hairdresser | Convulsions, typhoid fever |
| 14 | 45 „ | — | 1 | W. of draper's assist. | Small-pox confl. 6 dys.,(vaccin.) |
| 16 | 68 „ | 1 | — | Formerly a groom | Dysentery and anasarca |
| 26 | 70 „ | 1 | — | Agricl. labourer | Valvular dis. of heart, anasarca |
| 24 | 3wks | 1 | — | S. of dressmaker | Bronchitis |
| 26 | 12 yrs | — | 1 | D. of labourer | Meningitis |
| 29 | 36 „ | 1 | — | Horse clipper | Exhaustion caused by constant intemperance+ |
| 31 | 23 „ | — | 1 | D. of retired seaman | Diphtheria |
| Nov. 4 | 66 „ | 1 | — | Labourer | Malignant tumour of side |
| 5 | 86 „ | — | 1 | Widow of pensioner | Bronchitis |
| 8 | 83 „ | — | 1 | Widow of labourer | Paralysis, diarrhœa |
| 9 | 9 „ | — | 1 | D. of labourer | Dis. of mesenteric glands, 8 yrs. diseased spine 5 yrs., abscesses 5 yrs., variola 3 days |
| 12 | 25 „ | — | 1 | D. of druggist | Phthisis 2 years |
| 18 | 8 „ | 1 | — | S. of labourer | Small-pox (not vaccinated) |
| 19 | 76 „ | 1 | — | Former. schoolmaster | Atonic apoplexy |
| 20 | 9 „ | — | 1 | D. of labourer | Small-pox (not vaccinated) |
| 21 | 62 „ | — | 1 | W. of seaman | Obstruction of bowels 9 days |
| 22 | 58 „ | 1 | — | Cooper | Intermittent fever 5 months, chronic enteritis, perforation |
| 21 | 50 „ | — | 1 | W. of cordwainer | Obstruction of bowels 8 days |
| 21 | 14 „ | — | 1 | D. of joiner | Dis. of the heart accompanied with water in the chest‡ |
| 23 | 18 „ | 1 | — | Waterman | Small-pox 14 days (not vaccin.) |
| 25 | 74 „ | — | 1 | Widow of waterman | Softening of brain, apoplexy atonic |
| 24 | 18 „ | 1 | — | Oil-mill labourer | Small-pox 19 days, pueumonia 10 days |
| Total .... | | 19 | 19 | = 38 | |

The number of births during the above named period was 52; viz. males 30, females 32.

* Cause of death not certified. + Certified by coroner. ‡ Certified after *post mortem*.

*Gainsborough Quarterly Medical and Meteorological Register.*

| Treatment commenced. | Diseases of Private Patients. | Diseases of Pauper Patients. | Barometric pressure at the temp. of 32° Fahr. | Temperat. Dry bulb. | Temperat. Wet bulb. | Direction of Wind. | Rain in 24 hours. | Ozone. (Schoen.) | Remarks. |
|---|---|---|---|---|---|---|---|---|---|
| Aug 28 | Ovaritis, dyspepsia [diarr. | | 29·737 | 54 | 49 | NW. | ·0 | — | |
| 29 | Bronchitis, lumbago, emphysema, colic, | | 29·723 | 68 | 58 | NW. | ·05 | 8 | Fine to end |
| 30 | Dysuria, uterine hæmorrhage, dysen. | | 29·555 | 59 | 54 | SW. | ·09 | — | of month |
| 31 | Congestion of brain, ozæna, gastralgia | Diarrhœa | 29·603 | 52 | 50 | SW. | ·20 | 9 | |
| Sept. 1 | Cephalalgia intermittens, anæmia, intercostal neuralgia | | 29·544 | 63 | 58 | SW. | ·0 | — | Showery till the 8th |
| 2 | Tonsillitis, hæmorrhoids | Anæmia | 29·773 | 60 | 55 | S. | ·25 | 10 | |
| 3 | Encephalitis, prurigo incontinentia, | Epilepsia, congestio cerebri | 29·584 | 64 | 62 | S. | ·60 | 3 | |
| 4 | Epilepsia, gastrodynia [pleurodynia | Strumous ophthalmia | 29·645 | 63 | 62 | SW. | ·06 | — | |
| 5 | | Bronchitis, emphysema, lumbago | 29·742 | 65 | 60 | SW. W. | ·14 | 5 | |
| 6 | [tation | Diarr., rheumat., pleurisy, emphysema | 29·771 | 58 | 54 | SW. S. | ·04 | 8 | |
| 7 | Faucitis, diarr., anæmia from hyperlac- | Paralysis | 29·686 | 58 | 56 | SW. | ·06 | 2 | Fine till the |
| 8 | Febricula, intertrigo, diarr., dysentery | | 29·750 | 65 | 63 | S. NW. | ·0 | — | 14th |
| 9 | Puerperal convulsions | | 29·854 | 61 | 56 | S. | ·0 | 10 | |
| 10 | Hooping cough, vermes, strophulus | Ophthalmia | 29·784 | 65 | 61 | S. | ·0 | 6 | |
| 11 | Diarrhœa, scarlatina [weather | Variola, diarrhœa | 30·020 | 68 | 63 | S. | ·0 | — | |
| 12 | Hyperlactation, syncopia from heat of | | 30·152 | 65 | 61 | S. | ·0 | 10 | |
| 13 | Lichen, bronchocele, diarrhœa | Constipatio | 30·109 | 64 | 61 | SE. | ·0 | 10 | |
| 14 | Hooping cough, colic, supersecretion of milk | Faucitis | 30·222 | 58 | 57 | NE. | ·0 | 10 | Fog |
| 15 | Choleraic diarrhœa, small-pox, œdema | Petechiæ from want of food, faucitis | 30·200 | 59 | 57 | NE. | ·0 | 10 | „ |
| 16 | Leucorrhœa, variola | Furuncle, neuralgia, hemicrania | 30·150 | 57 | 56 | SE. | ·0 | 10 | „ |
| 17 | | Icterus, carbuncle | 29·809 | 64 | 62 | SE. | ·30 | 10 | |
| 18 | Diphtheria, diarrhœa | | 29·866 | 60 | 56 | SW. | ·0 | — | Thn., lg. & rn. |
| 19 | Colic, ophthalmia neonatorum | Dysentery, colic, morbus coxæ | 30·192 | 58 | 54 | SW. N. | ·0 | 10 | |
| 20 | Strumous ophth., bronchitis, emphys. | Lumbago, dropsy, icterus, diarrhœa | 30·212 | 58 | 56 | N. | ·0 | 2 | |
| 21 | Intercostal neuralgia, scarlat., hoop. ch. | Neuralgia | 30·259 | 51 | 50 | NE. | ·0 | 10 | Fog |
| 22 | Herpes, tonsillitis, diarr., neuralgia | Stomatitis, anthrax, rheumatism | 29·869 | 59 | 56 | E. | ·48 | 10 | Rain |
| 23 | Constipation, tonsillitis, dysentery | | 29·588 | 64 | 61 | SE. | ·02 | 3 | |
| 24 | Diarrhœa, dropsy after scarlatina | Dysentery | 29·980 | 60 | 55 | W. | ·0 | — | Fine |
| 25 | Tonsillitis, diphth., prurigo, rheumat. | Diarrhœa | 30·457 | 56 | 53 | SW. | ·0 | — | „ |
| 26 | Gravel, rheumatism | Variola | 30·436 | 63 | 60 | W. | ·0 | — | „ |

| | | | | | | | | | |
|---|---|---|---|---|---|---|---|---|---|
| 27 | English cholera | | 30·245 | 61 | 59 | NW. | ·0 | — | „ |
| 28 | Variola, ecthy., eczema impetiginoides | | 30·199 | 57 | 52 | NW. | ·02 | 10 | „ |
| 29 | Febris, erythema, dysenteria, diphth. | Chlorosis | 30·091 | 55 | 53 | SE. | ·09 | 10 | „ |
| 30 | Congestio cerebri, lumbago, colica, dysentery | | 29·542 | 58 | 57 | SE. | ·40 | — | Heavy rain |
| Oct. 1 | Hæmatemesis vicariosa, tonsillitis, fu- | | 29·992 | 50 | 47 | SW. | ·25 | 10 | |
| 2 | Bronchitis, pleuritis [runcle, diarr. | Diarrhœa | 29·922 | 55 | 53 | SW. | ·0 | 10 | Fine |
| 3 | | [rheum. | 29·878 | 68 | 61 | SW. | ·0 | 10 | „ |
| 4 | Pityriasis [colic | Diarr., scabies, tonsillitis et faucitis, | — | 58 | 55 | S. | ·08 | — | „ |
| 5 | Bronchitis, hoop. cough, dysen., gonorr., | Emphys., catarrh, phthisis, strum. oph. | 29·672 | 50 | 48 | SW. | ·02 | — | „ |
| 6 | Erysip., diarr., dysen., anæmia, dentitis | Diarrhœa, erythema nodosum, variola, | 29·943 | 48 | 46 | W. | ·0 | — | „ |
| 7 | Diphtheria, diarr., hoop. cough, gout, dysentery, varioloid, phthisis | Herpes, lumbago [lichen | 29·259 | 54 | 51 | S. NW. | ·02 | — | „ |
| 8 | Phthisis, neuralgia [dialgia | Faucitis, diarrhœa, rheumatism | 29·529 | 48 | 44 | NW. | ·0 | — | |
| 9 | Pneum., scarlat. anginosa, faucitis, car- | Dysentery, faucitis | 29·846 | 43 | 41 | NW. | ·06 | 10 | Boisterous |
| 10 | Dysen., hoop. cough, faucitis & tonsil- | Dyspepsia | 29·469 | 51 | 49 | SE. | ·32 | — | |
| 11 | Lichen [litis | | 29·498 | 45 | 45 | SE. | ·21 | — | |
| 12 | Hoop. cough, bronchitis & emphysema | Faucitis, tonsillitis | 29·853 | 48 | 47 | SW. | ·25 | 2 | |
| 13 | Abortio, vertigo | | 29·898 | 56 | 56 | NW. | ·0 | — | |
| 14 | Anæmia | Faucitis, variola, bronchitis, diarrhœa | 30·091 | 57 | 56 | NW. SW. | ·0 | — | Fine |
| 15 | Menorr., epilepsia, amenorr., paralysis | Neuralgia, variola, bronchitis | 30·027 | 58 | 57 | SW. | ·0 | 3 | |
| 16 | Erysip., diarr., atrophia, purulent opht. | | 29·799 | 57 | 56 | SW. | ·0 | — | |
| 17 | Bronchitis, incipient paralysis | Carbuncle, lumbago | 29·777 | 55 | 51 | SE. NW. | ·0 | — | |
| 18 | „ , intertrigo, faucitis with exanthem | Hæmatemesis | 29·902 | 45 | 44 | NW. | 1·15 | 10 | Rain and fog |
| 19 | Diarrhœa, intercostal neuralgia | | 29·877 | 48 | 46 | N. | ·88 | 10 | the end of |
| 20 | Dyspepsia, orchitis | Erysipelas, mania | 29·763 | 53 | 53 | N. | ·04 | 2 | the month |
| 21 | Tonsillitis | Bronchitis, diarrhœa, epilepsy, colic | 29·894 | 47 | 46 | N. | ·0 | 2 | |
| 22 | | Laringismus stridulus, trichonosis | 30·030 | 51 | 50 | NE. | ·0 | — | |
| 23 | Bronchitis, anæmia from prolonged | Erysip., sclerotitis, bronchitis, variola | 30·117 | 48 | 48 | NE. | ·0 | — | |
| 24 | Cerebral congestion, diarr. [lactation | Phthisis, rheum., bronchitis, pleuritis | 30·122 | 49 | 48 | NE. | ·0 | — | |
| 25 | Erysipelas | Conjunctivitis, diarrhœa, variola | 30·404 | 48 | 46 | NW. | ·0 | — | |
| 26 | Faucitis, menorrhagia | Gastrodynia | 30·395 | 49 | 48 | NW. | ·0 | — | |
| 27 | Diphtheria, faucitis | Porrigo, pneumonia, dysentery | 30·306 | 47 | 46 | S. | ·52 | — | |
| 28 | | Variola | 30·081 | 49 | 49 | N. | ·09 | — | |
| 29 | Diphtheria | Variola, leucorrhœa | 30·364 | 41 | 39 | N. | ·0 | — | |
| 30 | Faucitis, tonsillitis, otorrhœa, catarrh | Tonsillitis | 30·624 | 38 | 38 | N. | ·0 | — | |
| 31 | „ „ , scarlatina anginosa | Variola | 30·693 | 45 | 43 | N. | ·0 | — | |

The term *faucitis* I have applied to the cases of diphtheria when in the first stage; *i.e.* before any white deposit is found.

| Treatment commenced. | Diseases of Private Patients. | Diseases of Pauper Patients. | Barometric pressure at the temp. of 32° Fahr. | Temperat. | | Direction of Wind. | Rain in 24 hours. | Ozone. (Schoen.) | Remarks. |
|---|---|---|---|---|---|---|---|---|---|
| | | | | Dry bulb. | Wet bulb. | | | | |
| Nov. 1 | Faucitis, tonsillitis, diphth., paralysis | Arthritis, diarrhœa, paralysis | 30·535 | 47 | 46 | N. | ·04 | — | Dense fogs and showers to the end |
| 2 | Diphtheria, dyspepsia, herpes, dropsy | Scabies, impetigo | 30·453 | 48 | 47 | SE. | ·0 | — | |
| 3 | Orchitis, neuralgia, diarrhœa | Herpes, bronchitis, paralysis | 30·404 | 46 | 45 | W. | ·0 | — | |
| 4 | Faucitis, dysentery, variola | Bronchitis, faucitis, variola | 30·357 | 47 | 46 | N. | ·04 | — | |
| 5 | „ , diphtheria, mammitis, bronchitis, varioloid | | 30·226 | 46 | 44 | W. | ·09 | 6 | |
| 6 | Faucitis, impetigo, gout | Rheumatism | 30·404 | 40 | 37 | W. | ·01 | — | |
| 7 | Diphtheria, neuralgia, variola | | 30·483 | 40 | 39 | N. | ·0 | — | |
| 8 | Colic | Lumbago,asthma,herpes, fistula inpe- | 30·365 | 43 | 41 | N. | ·04 | — | |
| 9 | Faucitis, leucorrhœa | Bronchitis, variola, faucitis [rineo | 30·490 | 41 | 40 | N. | .01 | — | |
| 10 | Faucitis | Scabies,bronchitis,variola,rheumatism | 30·439 | 38 | 37 | W. | ·01 | — | |
| 11 | „ , diphtheria, porrigo scutulata | Lichen, variola, faucitis | 30·384 | 40 | 39 | W. | ·0 | 8 | |
| 12 | „ „ | Meningitis, variola, catarrh, hepatitis | 30·196 | 37 | 36 | SE. | ·0 | 4 | |
| 13 | Metritis, faucitis, diphth., periostitis | Dysentery, catarrh, variola | 29·790 | 32 | 32 | E. | ·0 | 10 | |
| 14 | Faucitis, diphtheria, catarrh, diarrhœa | Catarrh, dyspepsia, jaundice, apoplexy | 29·503 | 46 | 42 | E. | ·02 | 10 | |
| 15 | „ „ , gravel, variola, piles | Valvular disease of heart, variola | 29·819 | 40 | 35 | E. | ·0 | 7 | |
| 16 | Erysipelas, scabies, diarrhœa | | 29·634 | 35 | 32 | E. | ·0 | 2 | |
| 17 | Faucitis et adenitis, vermes | Laryngismus stridulus, rheumatism | 29·618 | 38 | 35 | E. | ·0 | 3 | |
| 18 | Epileptoid, bronchitis | Diarrhœa | 29·741 | 34 | 32 | N. | ·0 | — | |
| 19 | Diarrhœa, intercostal neuralgia | Colic, ramol. cerebri | 29·826 | 32 | 32 | NW. | ·02 | — | |
| 20 | Dyspepsia, orchitis | Orchitis, paralysis, variola | 29·934 | 35 | 34 | NW. | ·0 | — | |
| 21 | Tonsillitis | Variola | 30·148 | 35 | 34 | N. | ·0 | — | |
| 22 | [lactation | | 30·228 | 25 | 24 | N. | ·0 | — | |
| 23 | Bronchitis, anæmia from prolonged | Adenitis | 30·089 | 18 | 17 | N. | ·0 | — | |
| 24 | Diarrhœa, cerebral congestion | Eczema, bronchitis, gravel | 29·934 | 21 | 20 | N. | ·01 | 2 | |
| 25 | Erysipelas | Herpes zoster, rubeola, faucitis | 29·521 | 39 | 38 | SE. | ·35 | 8 | |
| 26 | Faucitis, menorrhagia | Bronchitis | 29·259 | 48 | 48 | SE. | ·03 | 10 | |

by no means unhealthy months and had rather less of the ordinary autumnal complaints. During November there has been a considerable amount of disease, still not particularly fatal."

*Lerwick.*—Dr. Spence writes :—" September, especially the latter half of it, was very changeable, boisterous, and wet. The beginning of October was remarkably stormy. On the 1st a vessel in the roadstead was struck by lightning and seriously damaged; and on the 7th it blew a perfect hurricane from the south east. Easterly winds prevailed during the middle of October, which was variable and wet, and there was a slight fall of snow on the 28th. The first few days of November were foggy and rainy; the remainder of the month was seasonable with prevailing northerly winds, and the ground was covered with snow from the 18th to the 22nd inclusive. Comparing this quarter with the corresponding period last year, there was a striking rarity of diarrhœa, and a decided prevalence of influenza. Scarlet fever occurred in the second week of September, and about the same time I saw a case of dropsy, with desquamation of the cuticle, which I think must have been scarlatinal. There was one case of modified smallpox, imported from Leith, but the disease did not spread. Ordinary catarrh was less prevalent than usual. Influenza prevailed epidemically during the last half of the quarter, not only in Lerwick, but as far as I could learn, throughout the whole of the Shetland Isles. Influenza is generally said to spare infants; but in this epidemic children from six to twelve months were almost without exception attacked with bronchitis of an apparently alarming severity, though in my own practice there were no fatal cases. Otitis was a very common complication of the influenza. I saw one case where there was an eruption slightly resembling scarlatina, and in several cases there were aphthæ in the mouth, which might have deceived careless observers into a belief in the presence of diphtherite. The case of gastric fever (by which I mean the mild form of typhoid or gastro-enteric fever) as well as the case of puerperal fever, both occurred in an undrained locality with much putrifying animal matter in the neighbourhood. The number of deaths was eleven; the average age at death was forty-five, there being four below five, and five above seventy. The causes, where I could ascertain them, were—paralysis, two; infantile convulsions, one; valvular disease of heart, three; chronic bronchitis, one; jaundice, one; debility from want of breast-milk, one; extensive burn of chest, one."

